# Supplementary material for: ABCG2 transports anticancer drugs via a closed-to-open switch
Source: Nat Commun. 2020 May 8;11:2264. doi: 10.1038/s41467-020-16155-2 (PMC7210939; doi:10.1038/s41467-020-16155-2)
Supplement: Supplementary file 1 — Supplementary Information [file 41467_2020_16155_MOESM1_ESM.pdf]

ABCG2 transports anticancer drugs via an unexpected closed-to-open switch

Benjamin J. Orlando and Maofu Liao

Supplementary Information:
Supplementary Figures 1-10
Supplementary Tables 1-3

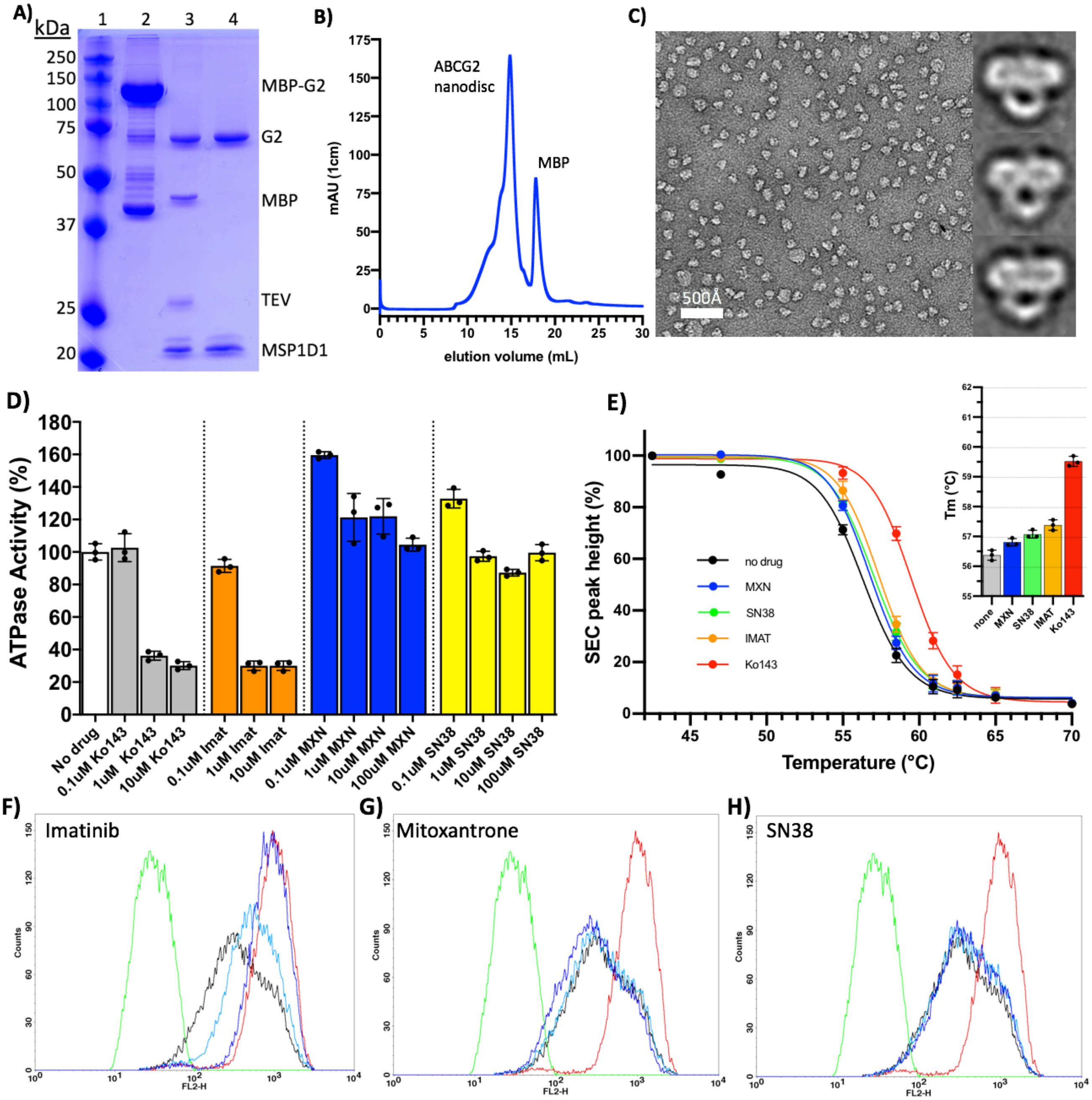

Supplementary Figure 1. Biochemical Characterization of ABCG2

A) Reducing SDS-PAGE of ABCG2 fractions during purification. Lane 1: molecular weight marker. Lane 2: MBP-ABCG2 after amylose affinity purification. Lane 3. ABCG2 after nanodisc incorporation and TEV cleavage. Lane 4: final gel-filtration purified ABCG2-nanodiscs. The experiment was repeated once. Source data are provided as a Source Data file. **B)** Gel-filtration profile of ABCG2-nanodiscs. **C)** Representative negative-stain electron micrograph and 2D class averages of purified ABCG2-nanodiscs. The experiment was repeated once. **D)** ATPase activity of nanodisc reconstituted ABCG2 in the presence of different inhibitors and substrates. Bars represent the average of three independent measurements with SEM. **E)** Thermal shift analysis of detergent solubilized ABCG2 in the presence of different transport substrates and inhibitors. All compounds were included at a concentration of 20uM. Experiments were repeated in triplicate with three independent replicates. Inset shows the mean melting temperature among three experiments with standard deviation. Source data are provided as a Source Data file. **F-H)** 5D3 antibody shift flow cytometry histograms in the presence of imatinib (**F**), MXN (**G**), or SN38 (**H**). Green histogram indicates cells stained with only secondary PE-antibody and no 5D3. Black histogram indicates cells without added compound. Red histogram represents cells in the presence of 5uM Ko143. Light blue and dark blue histograms represent cells incubated with 1uM or 10uM of the indicated compound. Each experiment was completed once. Gating strategy is provided as a Source Data file.

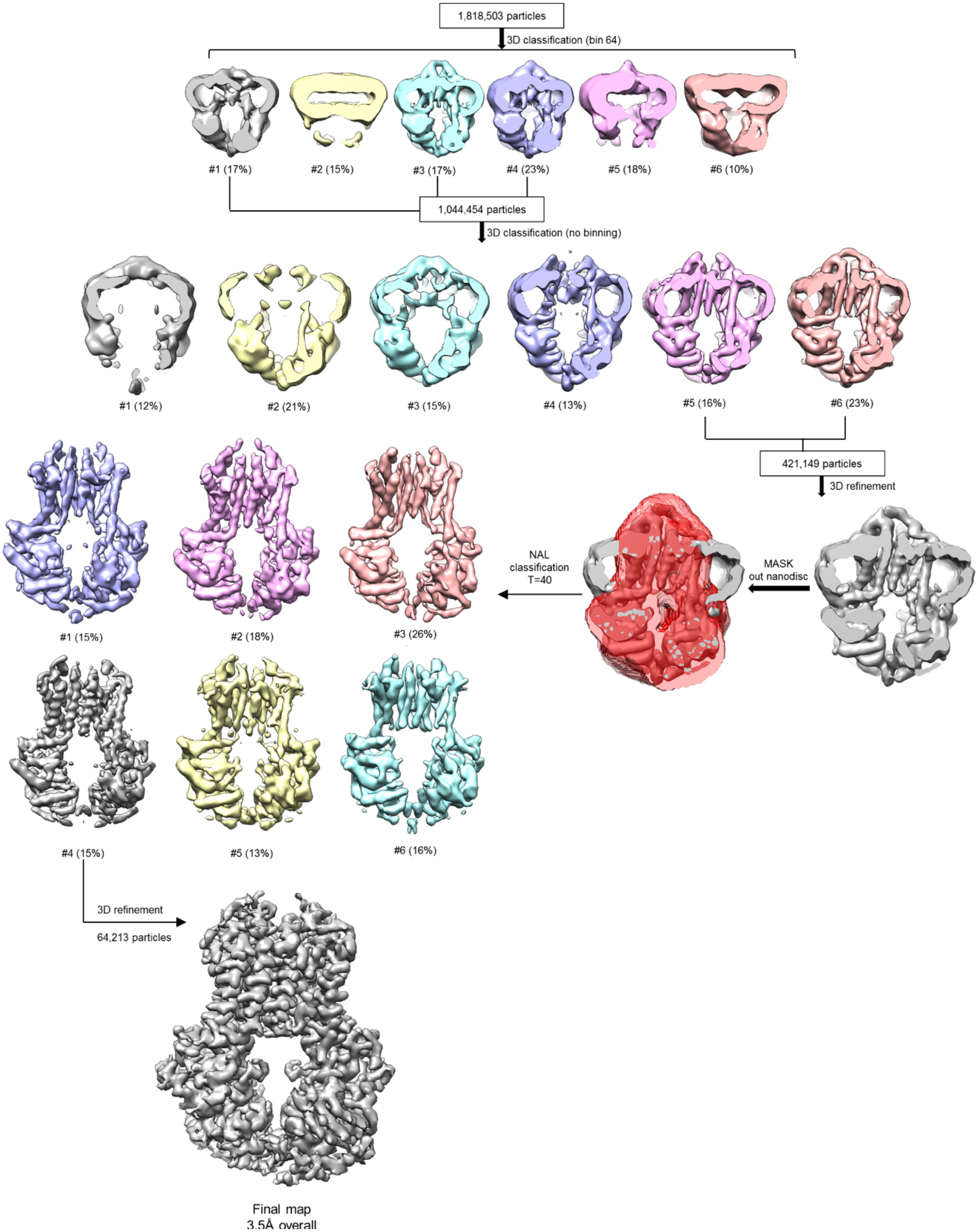

Supplementary Figure 2. Cryo-EM Data Processing of apo-closed ABCG2.
Flowchart showing 3D classification and refinement scheme of apo-closed ABCG2.

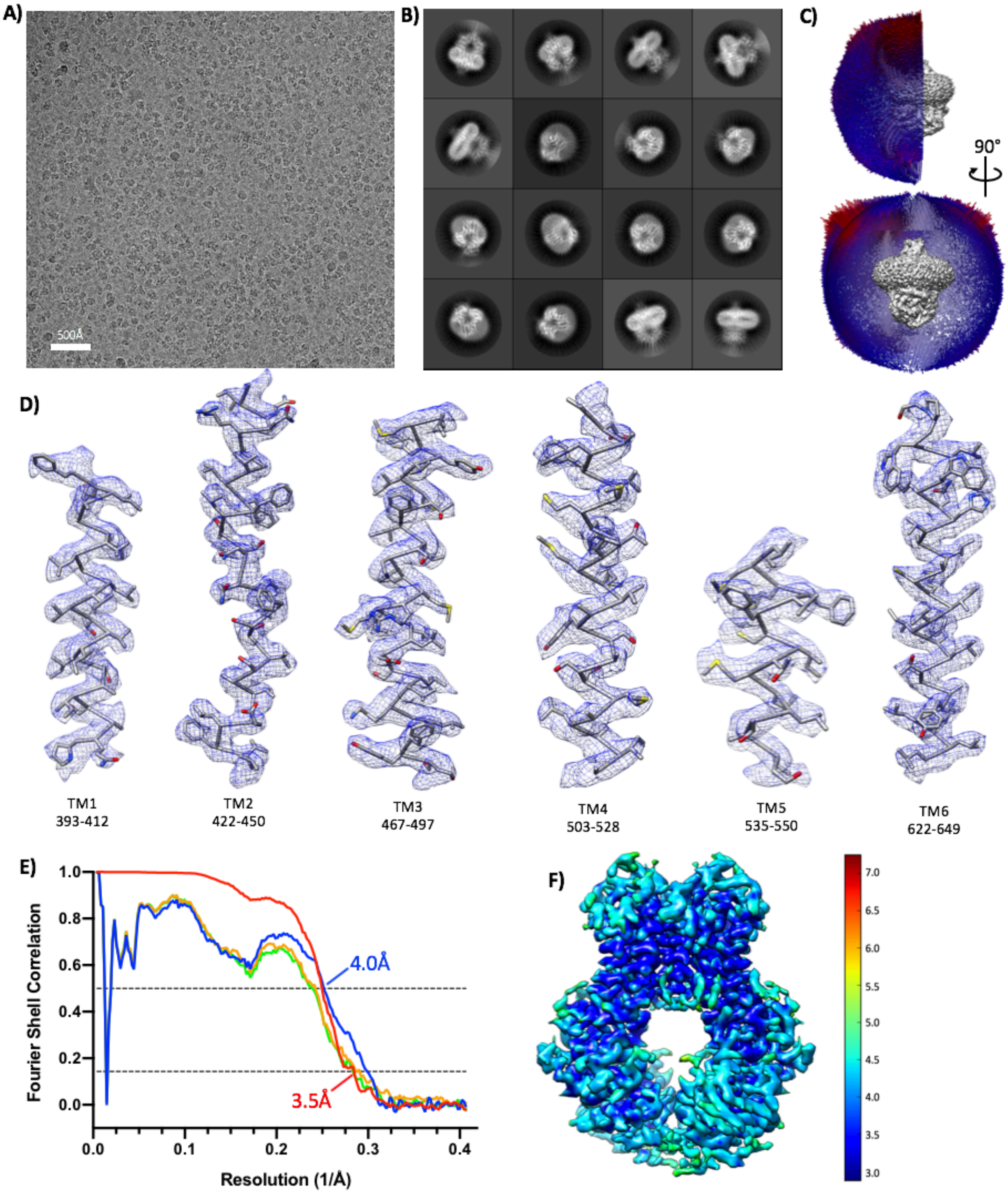

Supplementary Figure 3. Cryo-EM Data Analysis of apo-closed ABCG2

A) Representative electron micrograph of apo ABCG2-nanodiscs. The experiment was repeated once. **B)** Representative 2D class averages of ABCG2 nanodiscs. **C)** Angle distribution of final reconstruction. **D)** Cryo-EM map for TM helices with refined model. **E)** FSC curves. Red: half map 1 vs. half map 2. Blue: Model vs. final map. Green: model refined against half map 1 vs. half map 1. Orange: model refined against half map 1 vs. half map 2. **F)** Local resolution plot of final map.

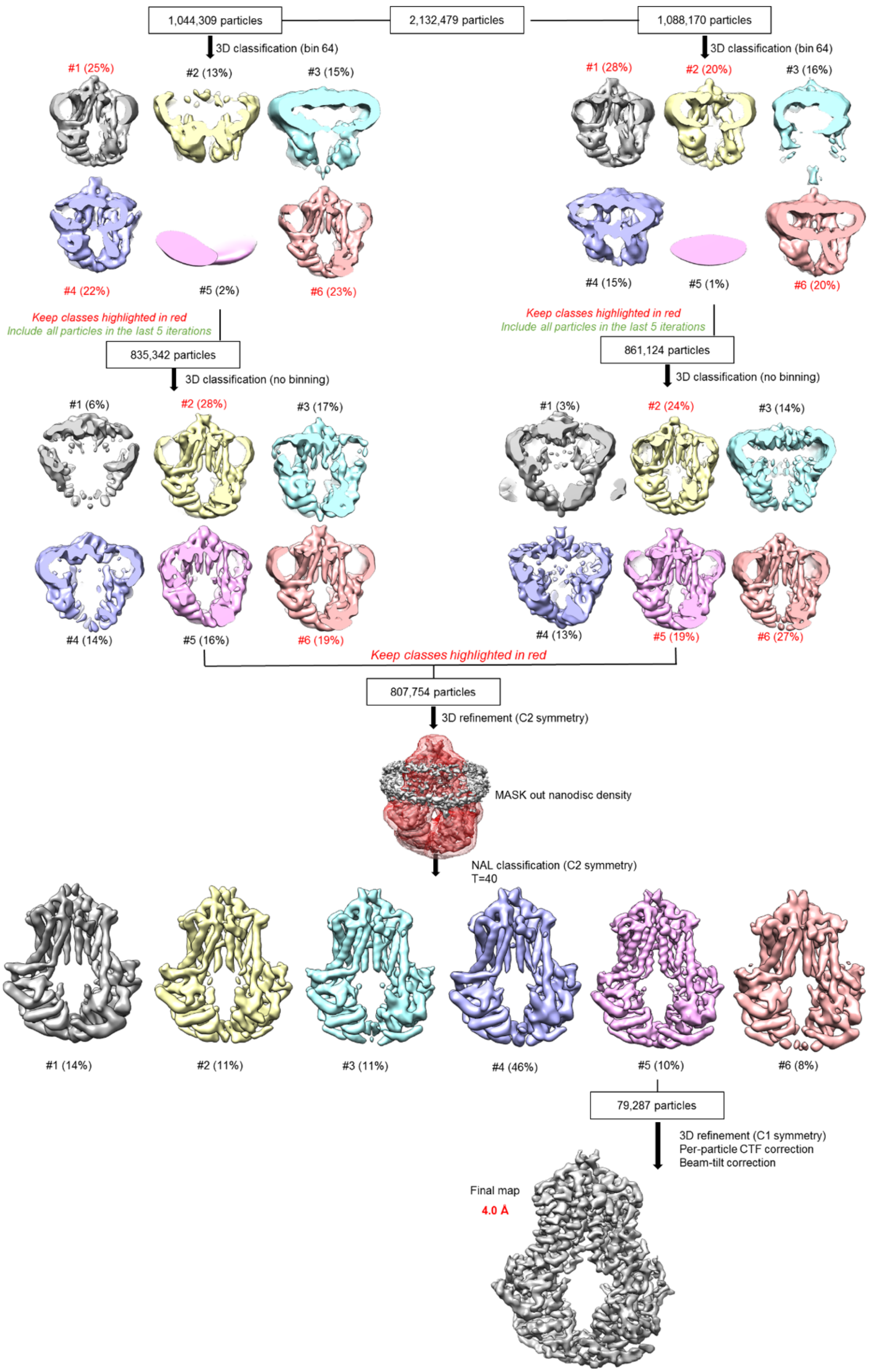

Supplementary Figure 4. Cryo-EM Data Processing of ABCG2-imatinib.
Flowchart showing 3D classification and refinement scheme of ABCG2-imatinib

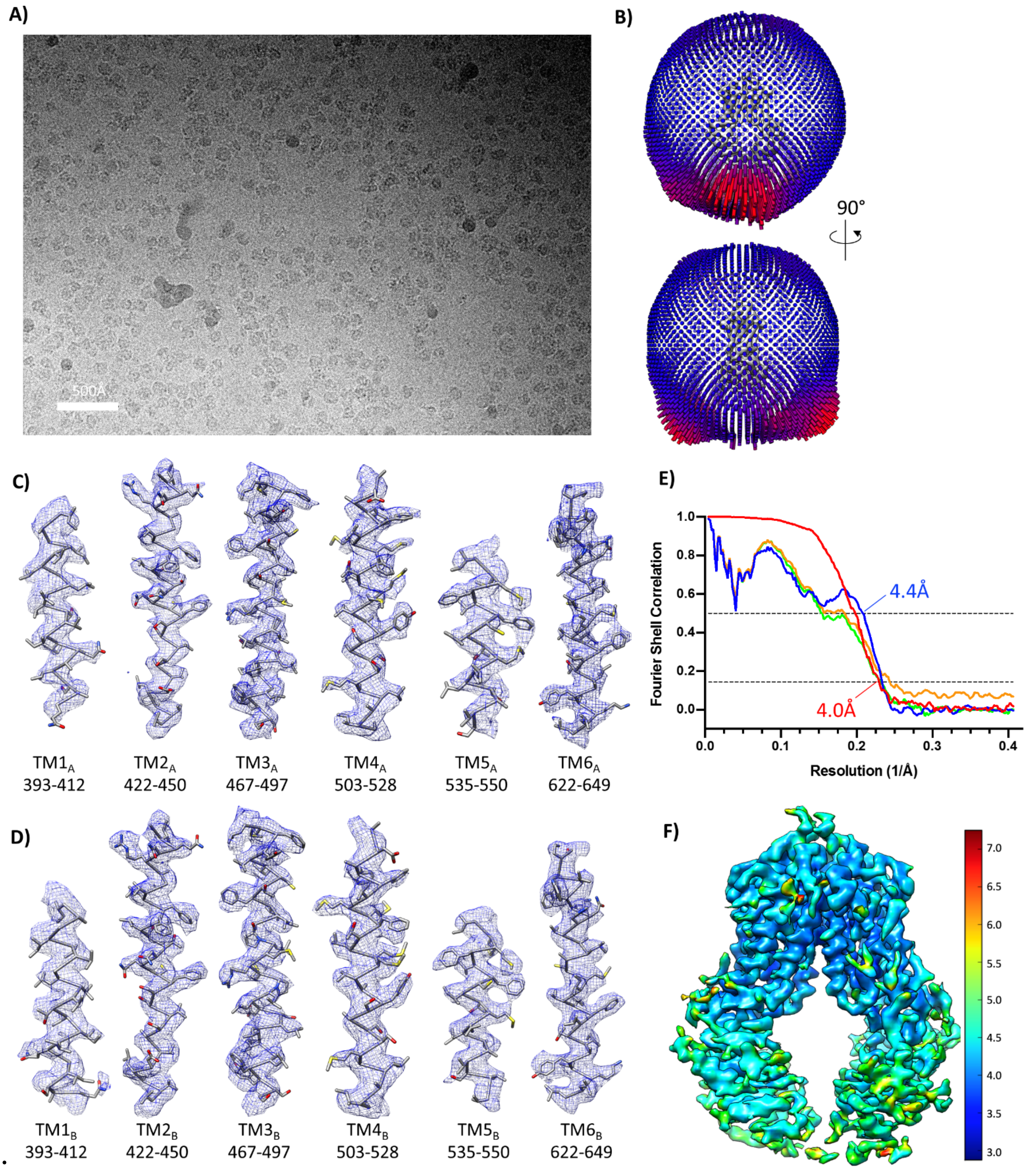

Supplementary Figure 5. Cryo-EM Data Analysis of ABCG2-Imatinib
A) Representative cryo electron micrograph of ABCG2-nanodiscs with imatinib. **B)** Angle distribution of final reconstruction. The experiment was repeated once. **C)** Cryo-EM map for TM helices of monomer A. **D)** Cryo-EM map for TM helices of monomer B. **E)** FSC curves for final reconstruction. Red: half map 1 vs. half map 2. Blue: Model vs. final map. Green: model refined against half map 1 vs. half map 1. Orange: model refined against half map 1 vs. half map 2. F) Local resolution plot of final ABCG2-imatinib map.

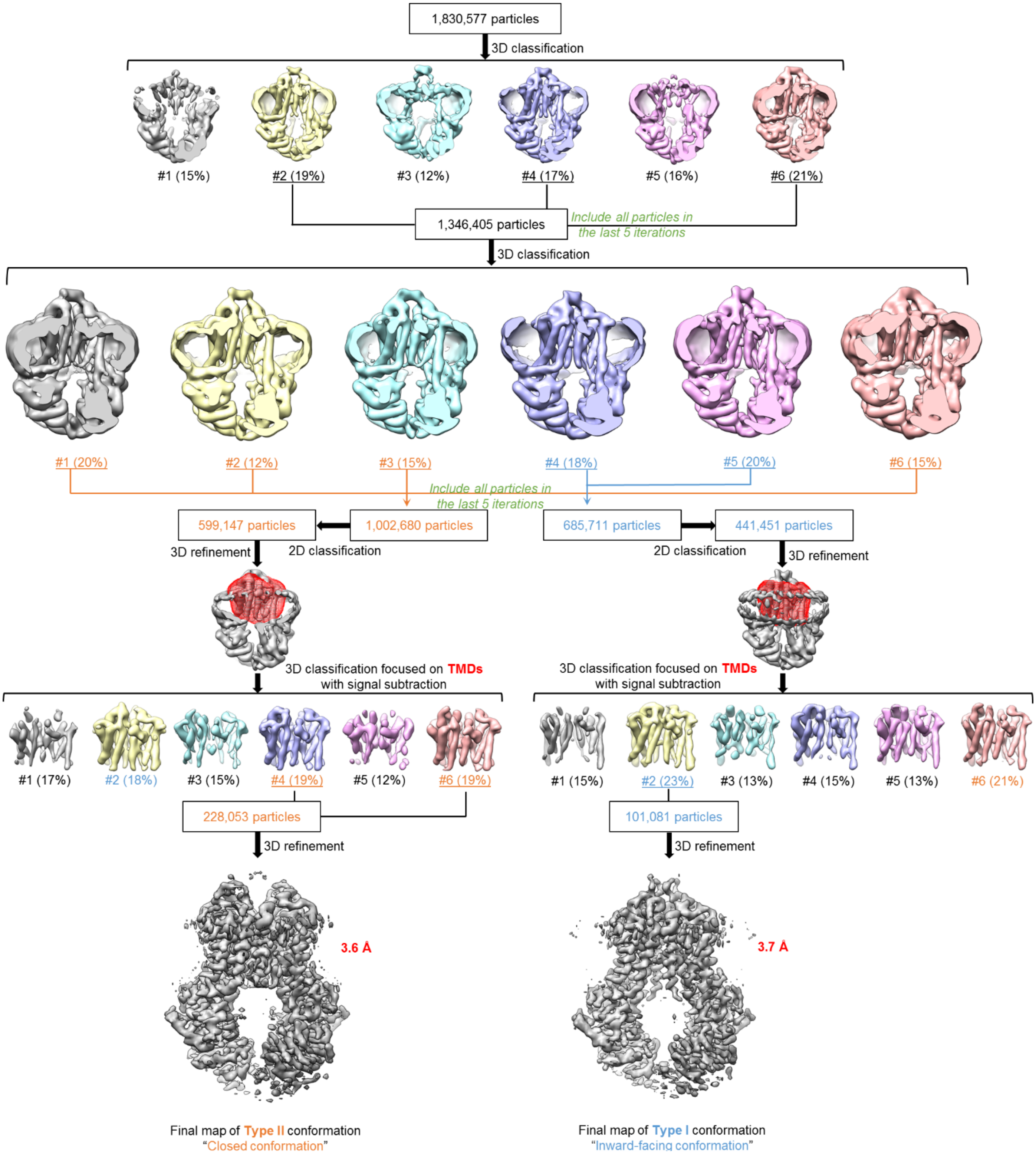

Supplementary Figure 6. Cryo-EM Data Processing of ABCG2-MXN
Flowchart showing 3D classification and refinement scheme of ABCG2-MXN.

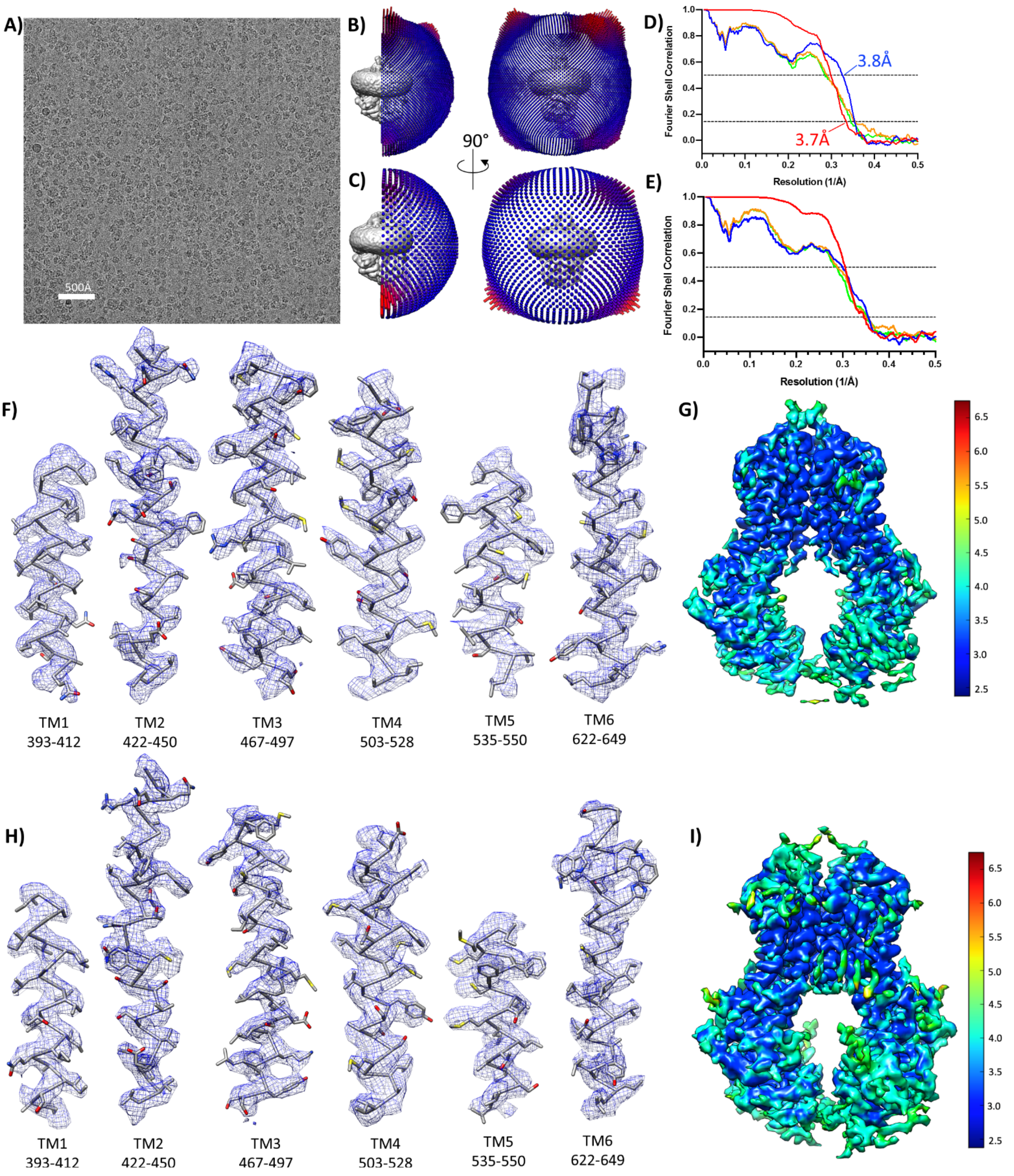

Supplementary Figure 7. Cryo-EM Data Analysis of ABCG2-MXN

A) Representative electron micrograph of ABCG2-nanodiscs with mitoxantrone. The experiment was repeated once. **B)** Angle distribution of final inward facing reconstruction. **C)** Angle distribution of final occluded reconstruction. **D)** FSC curves for inward facing conformation. Red: half map 1 vs. half map 2. Blue: Model vs. final map. Green: model refined against half map 1 vs. half map 1. Orange: model refined against half map 1 vs. half map 2. **E)** FSC curves for occluded conformation. Coloring is the same as **D**. **F)** Cryo-EM map for TM helices in inward facing conformation. **G)** Local resolution plot of inward facing conformation. **H)** Cryo-EM map for TM helices in occluded conformation. **I)** Local resolution plot of occluded conformation.

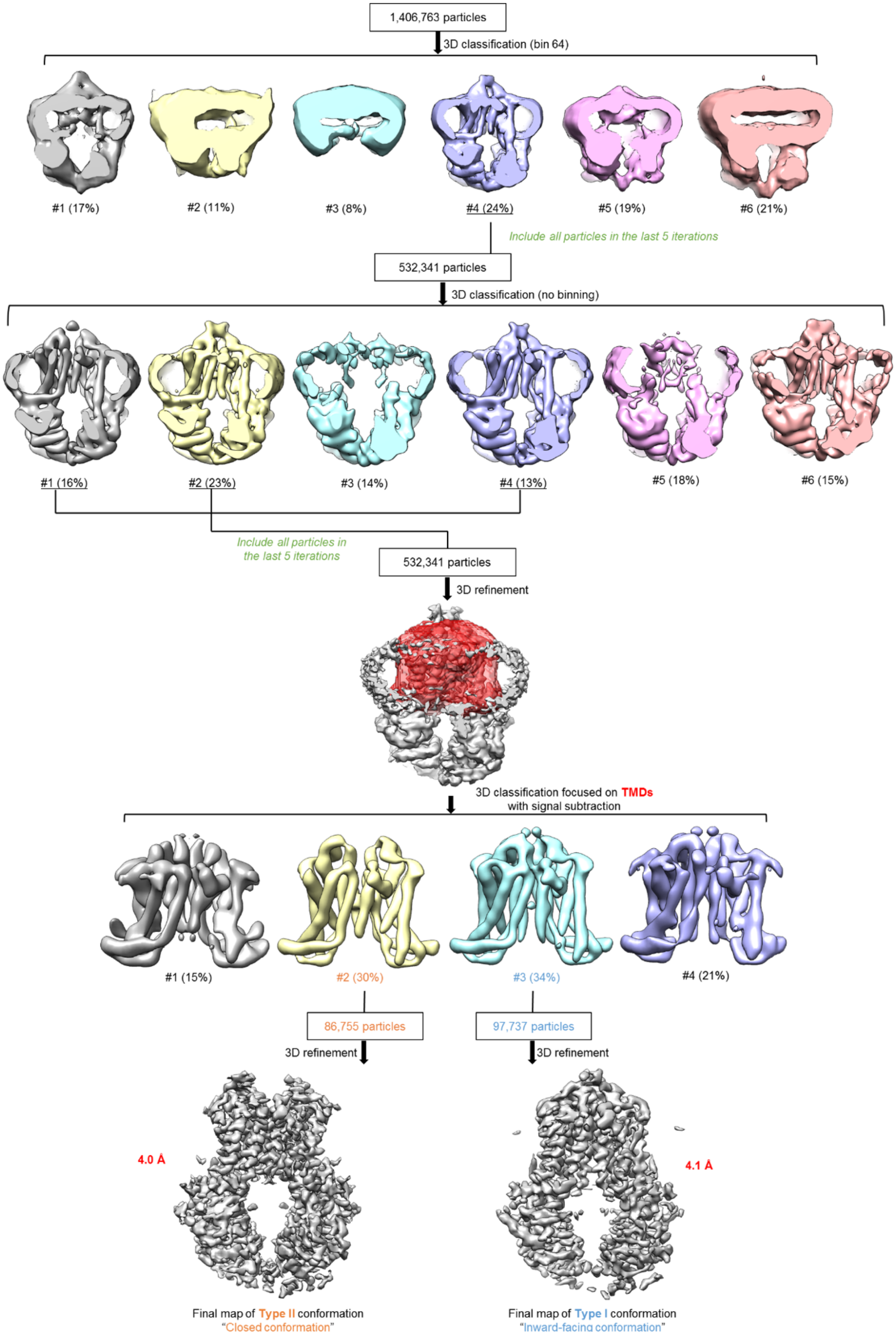

Supplementary Figure 8. Cryo-EM Data Processing of ABCG2-SN38
Flowchart showing 3D classification and refinement scheme of ABCG2-SN38..

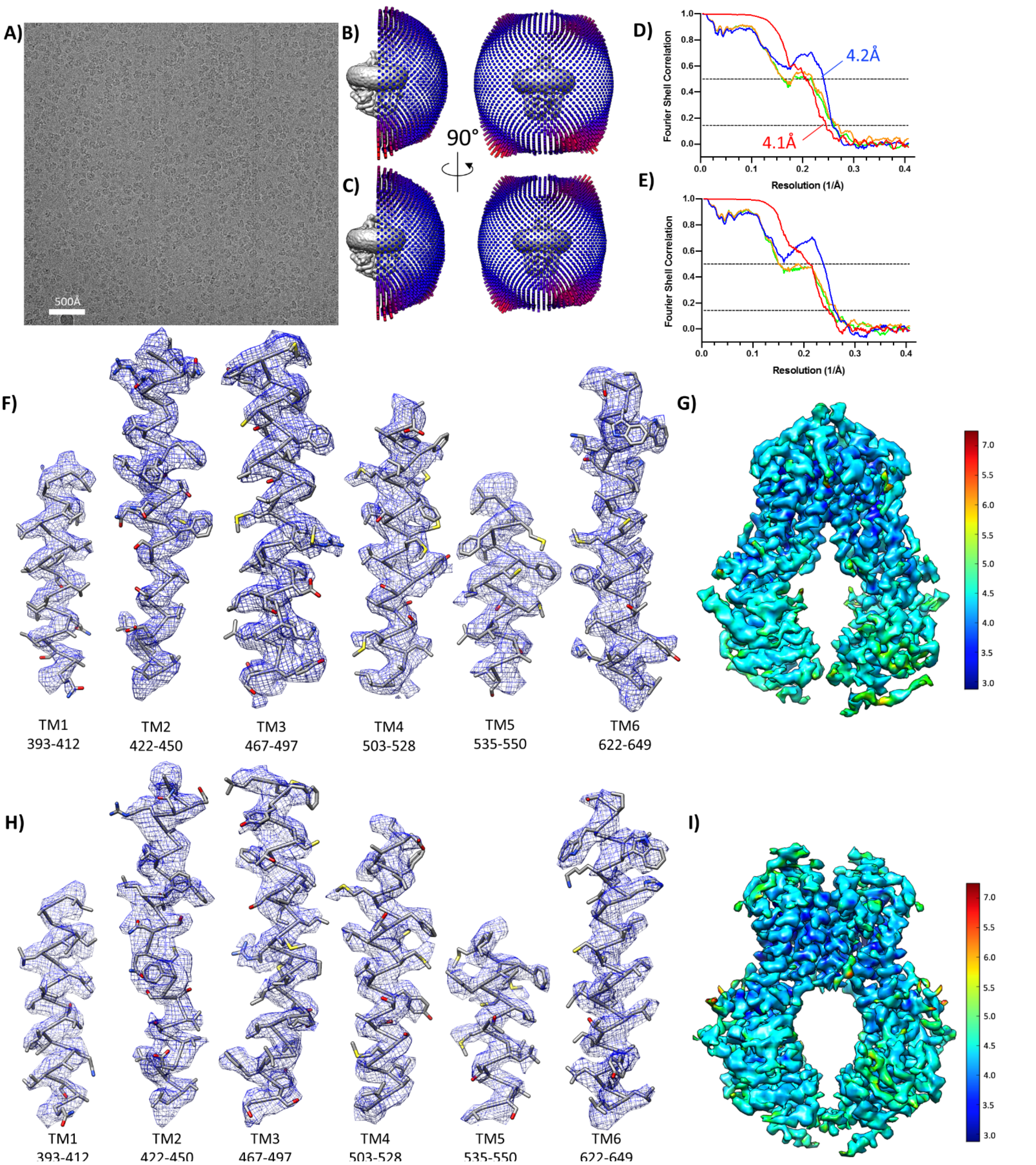

Supplementary Figure 9. Cryo-EM Data Analysis of ABCG2-SN38

A) Representative cryo electron micrograph of ABCG2-nanodiscs with SN38. The experiment was repeated once. **B)** Angle distribution of final inward facing reconstruction. **C)** Angle distribution of final occluded reconstruction. **D)** FSC curves for inward facing conformation. Red: half map 1 vs. half map 2. Blue: Model vs. final map. Green: model refined against half map 1 vs. half map 1. Orange: model refined against half map 1 vs. half map 2. **E)** FSC curves for occluded conformation. Coloring is the same as **D**. **F)** Cryo-EM map for TM helices in inward facing conformation. **G)** Local resolution plot of inward facing conformation. **H)** Cryo-EM map for TM helices in occluded conformation. **I)** Local resolution plot of occluded conformation.

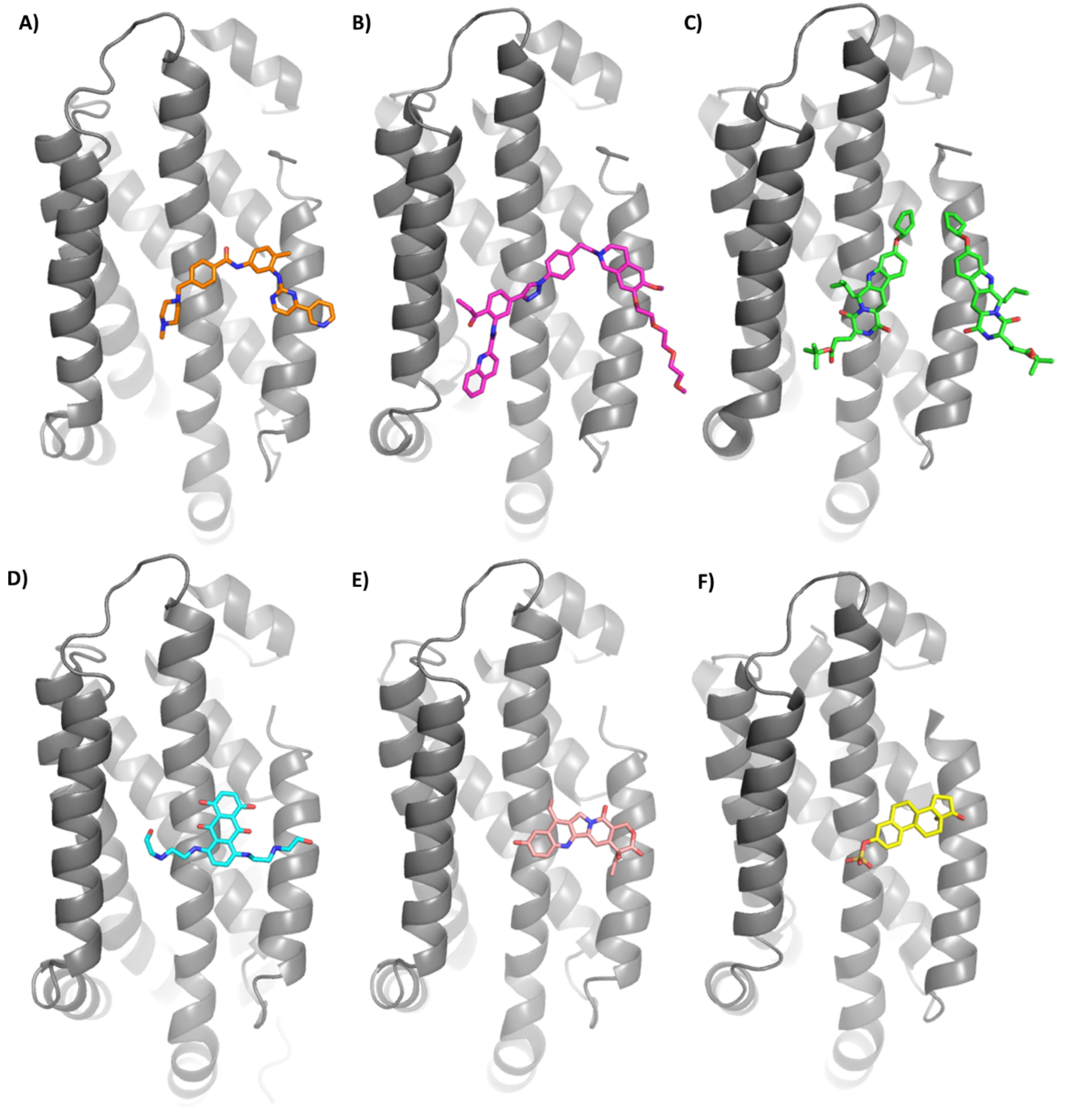

Supplementary Figure 10. Comparison of drug binding poses

Shown are the drug binding poses observed in all inward facing cryoEM structures of ABCG2 determined to date. Structures are oriented to show a single monomer of ABCG2 as viewed from the 2-fold dimer axis. On the top row are molecules that inhibit ABCG2 ATPase activity including imatinib (**A**), MB136 (PDBid 6FEQ) (**B**), and the Ko143 derivative MZ29 (PDBid 6ETI) (**C**). On the bottom row are known transport substrates of ABCG2 including MXN (**D**), SN38 (**E**), and estrone-3-sulfate (E_1S) (PDBid 6HCO) (**F**). All of the inhibitors and substrates bind in largely overlapping binding pockets, with MB136 and MZ29 occupying the largest volume at the dimer interface.

Supplementary Table 1. Cryo-EM data collection and processing statistics.

	ABCG2 Apo	ABCG2 Mitoxantrone		ABCG2 SN38		ABCG2 Imatinib
	Closed<br>PDBid: 6VXF<br>EMDB: 21436	Inward<br>PDBid: 6VXI<br>EMDB: 21438	Closed<br>EMDB: 21439	Inward<br>PDBid: 6VXJ<br>EMDB: 21440	Closed<br>EMDB: 21441	Inward<br>PDBid: 6VXH<br>EMDB: 21437
**Data collection and processing**						
Microscope	Polara	Polara		Polara		Titan Krios
Detector	K2 Summit	K2 Summit		K2 Summit		K3
Magnification	31,000	31,000		31,000		105,000
Voltage (kV)	300	300		300		300
Electron exposure (e–/Å^2)	52	52		52		66
Defocus range (μm)	1.0-2.5	1.0-3.0		1.0-3.0		1.0-3.0
Pixel size (Å)	1.23	1.23		1.23		0.85
Symmetry imposed	C2	C2		C2		C1
Initial particle images (no.)	1,818,503	1,830,577		1,406,763		2,132,479
Final particle images (no.)	64,213	101,081	228,053	97,737	86,755	79,287
Map resolution (Å)	3.5	3.7	3.6	4.1	4.0	4.0
FSC threshold	0.143	0.143	0.143	0.143	0.143	0.143
Map resolution range (Å)	2.5-5.0	2.5-5.0	2.5-5.0	3.0-6.0	3.0-6.0	3.0-6.5
**Refinement**						
Initial model used (PDBid)	6ETI	6ETI		6ETI		6ETI
Model resolution (Å)	4.0	3.8		4.2		4.4
FSC threshold	0.5	0.5		0.5		0.5
Map sharpening B factor (Å^2)	-150	-200		-260		-214
Model composition						
Non-hydrogen atoms	8786	8898		8895		8879
Protein residues	1130	1132		1132		1130
Ligands	0	1		1		1
Lipid	0	2		2		2
B factors (Å^2)						
Protein	51.1	55.8		89.8		94.4
Ligand	-	31.2		55.0		46.6
R.m.s. deviations						
Bond lengths (Å)	0.009	0.008		0.007		0.006
Bond angles (°)	1.034	1.135		1.181		1.204
Validation						
MolProbity score	1.80	2.13		2.30		1.82
Clashscore	4.68	6.40		6.68		5.13
Poor rotamers (%)	1.3	2.1		3.32		0.31
Ramachandran plot						
Favored (%)	92.1	90.3		90.5		89.9
Allowed (%)	7.7	9.5		9.5		9.9
Disallowed (%)	0.2	0.2		0.0		0.2

Supplementary Table 2. Distances between mutants in disulphide crosslinking assay.
Shown are the distances between residues 534-534 or 537-537 of opposite ABCG2 monomers in different conformational states. Distances are measured from the Cβ carbon of the respective side-chain. "Apo-closed" corresponds to the apo ABCG2 structure determined in this study. "Inward facing" corresponds to the inward facing MXN-bound ABCG2 structure determined in this study. "ATP bound" corresponds to the ATP-bound ABCG2 structure (PDBid 6HZM).

	Apo-closed	Inward Facing	ATP bound
**534-534**	13.8 Å	13.4 Å	5.6 Å
**537-537**	3.8 Å	19.4 Å	14.9 Å

Supplementary Table 3. Primers used in this study.
Shown below are the primers used for site-directed mutagenesis. Forward and reverse primers are listed for each individual point mutant.

Primer	Sequence
Cys603Ser-Forward	5'- AAACAATCCTAGTAACTATGCAAC – 3'
Cys603Ser-Reverse	5'- CCTGTTGCATTGAGTCCTG -3'
Val534Cys-Forward	5'- TCAGAGTGTGTGTTCTGTAGCAACACTTCTCATG - 3'
Val534Cys-Reverse	5'- CCTGCTGCTATGGCCAGT -3'
Ala537Cys-Forward	5'- GGTTTCTGTATGTACACTTCTCATGACCATCTGTTTTG -3'
Ala537Cys-Reverse	5'- ACACTCTGACCTGCTGCT -3'
