# Supplementary material for: ABCG2 transports anticancer drugs via a closed-to-open switch
Source: Nat Commun. 2020 May 8;11:2264. doi: 10.1038/s41467-020-16155-2 (PMC7210939; doi:10.1038/s41467-020-16155-2)
Supplement: Supplementary file 3 — Reporting Summary [file 41467_2020_16155_MOESM3_ESM.pdf]

nature research

Corresponding author(s): Maofu Liao

Last updated by author(s): Apr 9, 2020

Reporting Summary

Nature Research wishes to improve the reproducibility of the work that we publish. This form provides structure for consistency and transparency in reporting. For further information on Nature Research policies, see Authors & Referees and the Editorial Policy Checklist .

Statistics

For all statistical analyses, confirm that the following items are present in the figure legend, table legend, main text, or Methods section.

n/a	Confirmed	
[ ]	[x]	The exact sample size (n) for each experimental group/condition, given as a discrete number and unit of measurement
[ ]	[x]	A statement on whether measurements were taken from distinct samples or whether the same sample was measured repeatedly
[x]	[ ]	The statistical test(s) used AND whether they are one- or two-sided<br>*Only common tests should be described solely by name; describe more complex techniques in the Methods section.*
[x]	[ ]	A description of all covariates tested
[x]	[ ]	A description of any assumptions or corrections, such as tests of normality and adjustment for multiple comparisons
[ ]	[x]	A full description of the statistical parameters including central tendency (e.g. means) or other basic estimates (e.g. regression coefficient) AND variation (e.g. standard deviation) or associated estimates of uncertainty (e.g. confidence intervals)
[x]	[ ]	For null hypothesis testing, the test statistic (e.g. F, t, r) with confidence intervals, effect sizes, degrees of freedom and P value noted<br>*Give P values as exact values whenever suitable.*
[x]	[ ]	For Bayesian analysis, information on the choice of priors and Markov chain Monte Carlo settings
[x]	[ ]	For hierarchical and complex designs, identification of the appropriate level for tests and full reporting of outcomes
[x]	[ ]	Estimates of effect sizes (e.g. Cohen's d, Pearson's r), indicating how they were calculated

Our web collection on statistics for biologists contains articles on many of the points above.

Software and code

Policy information about availability of computer code

Data collection	Cryo-EM data was collected with SerialEM (3.7). Flow-cytometry data was collected with BD CellQuest Pro (5.2.1).
Data analysis	Phenix; Coot; MotionCor2; CTFFIND4; SAMUEL; SamViewer; SPIDER; RELION3.0; ResMap; bfactor; Chimera; BD CellQuest Pro

For manuscripts utilizing custom algorithms or software that are central to the research but not yet described in published literature, software must be made available to editors/reviewers. We strongly encourage code deposition in a community repository (e.g. GitHub). See the Nature Research guidelines for submitting code & software for further information.

Data

Policy information about availability of data

All manuscripts must include a data availability statement. This statement should provide the following information, where applicable:

- Accession codes, unique identifiers, or web links for publicly available datasets
- A list of figures that have associated raw data
- A description of any restrictions on data availability

The three-dimensional cryo-EM density maps of ABCG2 in nanodiscs have been deposited in the Electron Microscopy Data Bank under accession numbers EMD-21436 (apo), EMD-21437 (imatinib), EMD-21438 (MXN-inward), EMD-21439 (MXN-closed), EMD-21440 (SN38-inward), and EMDB-21441 (SN38-closed). Atomic coordinates for the atomic models of ABCG2 have been deposited in the Protein Data Bank under accession numbers 6VXF (apo), 6VXH (imatinib), 6VXI (MXN-inward), 6VXJ (SN38-inward). Correspondence and requests for materials should be addressed to M.L. (maofu_liao@hms.harvard.edu).

Field-specific reporting

Please select the one below that is the best fit for your research. If you are not sure, read the appropriate sections before making your selection.

Life sciences study design

All studies must disclose on these points even when the disclosure is negative.

Sample size	No statistical methods were used to predetermine sample size.
Data exclusions	No data were excluded from analyses.
Replication	All replicas of data produced similar results.
Randomization	This is not relevant to our study, because no grouping was needed.
Blinding	Investigators were not blinded to group allocation. It is not applicable to studies reported.

Reporting for specific materials, systems and methods

We require information from authors about some types of materials, experimental systems and methods used in many studies. Here, indicate whether each material, system or method listed is relevant to your study. If you are not sure if a list item applies to your research, read the appropriate section before selecting a response.

Materials & experimental systems

n/a	Involved in the study	
☐	☒	Antibodies
☐	☒	Eukaryotic cell lines
☒	☐	Palaeontology
☒	☐	Animals and other organisms
☒	☐	Human research participants
☒	☐	Clinical data

Methods

n/a	Involved in the study	
☒	☐	ChIP-seq
☐	☒	Flow cytometry
☒	☐	MRI-based neuroimaging

Antibodies

Antibodies used	Anti-BCRP clone 5D3 (R&D Systems product MAB995), goat-anti-mouse PE conjugated secondary (Sigma Aldrich product P9670)
Validation	Zhou, S., Schuetz, J. D., Bunting, K. D., Colapietro, A. M., Sampath, J., Morris, J. J., Lagutina, I., Grosveld, G. C., Osawa, M., Nakauchi, H., and Sorrentino, B. P. (2001) Nat. Med. 7, 1028–1034

Eukaryotic cell lines

Policy information about cell lines

Cell line source(s)	Freestyle 293F from Thermo Fisher Scientific
Authentication	Cell lines were not authenticated
Mycoplasma contamination	Cell lines were not tested for mycoplasma contamination
Commonly misidentified lines (See ICLAC register)	N/A

Flow Cytometry

Plots

Confirm that:

- ☒ The axis labels state the marker and fluorochrome used (e.g. CD4-FITC).
- ☒ The axis scales are clearly visible. Include numbers along axes only for bottom left plot of group (a 'group' is an analysis of identical markers).
- ☒ All plots are contour plots with outliers or pseudocolor plots.
- ☒ A numerical value for number of cells or percentage (with statistics) is provided.

Methodology

Sample preparation	Cells were grown on plates, trypsinized, and then stained for flow-cytometry.
Instrument	BD FacsCalibur
Software	BD CellQuest Pro v 5.2.1
Cell population abundance	A single cell line was used.
Gating strategy	Cell were gated first on FSC/SSC, followed by GFP expression level.

☒ Tick this box to confirm that a figure exemplifying the gating strategy is provided in the Supplementary Information.
